# Can biomarkers identified from the uterine fluid transcriptome be used to establish a noninvasive endometrial receptivity prediction tool? A proof-of-concept study

**DOI:** 10.1186/s12958-023-01070-0

**Published:** 2023-02-18

**Authors:** Aihua He, Hong Wu, Yangyun Zou, Cheng Wan, Jing Zhao, Qiong Zhang, Nenghui Liu, Donge Liu, Yumei Li, Jing Fu, Hui Li, Xi Huang, Tianli Yang, Chunxu Hu, Zhaojuan Hou, Yue Sun, Xin Dong, Jian Wu, Sijia Lu, Yanping Li

**Affiliations:** 1 Department of Reproductive Medicine, Xiangya Hospital, Central South University, 87 Xiangya Road, Changsha, 410000 Hunan Province China; 2 Clinical Research Center for Women’s Reproductive Health in Hunan Province, Changsha, 410000 Hunan China; 3 Department of Reproductive Medicine, Third Xiangya Hospital, Central South University, Changsha, 410013 Hunan China; 4 Department of ENT, Xiangya Hospital, Central South University, Changsha, 410000 Hunan China; 5 Department of Clinical Research, Yikon Genomics Company, Ltd., #301, Building A3, No. 218, Xinghu Street, Suzhou, 215123 Jiangsu China

**Keywords:** Endometrial receptivity, Window of implantation, Transcriptomic profiling, Machine learning, Random forest algorithm, Noninvasive biomarker

## Abstract

**Background:**

Embryo implantation in a receptive endometrium is crucial for successful pregnancy. Endometrial receptivity (ER) prediction tools based on endometrial transcriptome biomarkers by endometrial biopsy have been used to guide successful embryo implantation in in vitro fertilization (IVF) patients. However, no reliable noninvasive ER prediction method has been established, and one is greatly needed. We aimed to identify biomarkers from uterine fluid transcriptomic sequencing data for establishing noninvasive ER prediction tool and to evaluate its clinical application potential in patients undergoing IVF.

**Methods:**

The non-invasive RNA-seq based endometrial receptivity test (nirsERT) was established by analyzing transcriptomic profile of 144 uterine fluid specimens (LH + 5, LH + 7, and LH + 9) at three different receptive status from 48 IVF patients with normal ER in combination with random forest algorithm. Subsequently, 22 IVF patients who underwent frozen-thaw blastocyst transfer were recruited and analyzed the correlation between the predicted results of nirsERT and pregnancy outcomes.

**Results:**

A total of 864 ER-associated differentially expressed genes (DEGs) involved in biological processes associated with endometrium-embryo crosstalk, including protein binding, signal reception and transduction, biomacromolecule transport and cell-cell adherens junctions, were selected. Subsequently, a nirsERT model consisting of 87 markers and 3 hub genes was established using a random forest algorithm. 10-fold cross-validation resulted in a mean accuracy of 93.0%. A small cohort (*n* = 22) retrospective observation shows that 77.8% (14/18) of IVF patients predicted with a normal WOI had successful intrauterine pregnancies, while none of the 3 patients with a displaced WOI had successful pregnancies. One patient failed due to poor sequencing data quality.

**Conclusions:**

NirsERT based on uterine fluid transcriptome biomarkers can predict the WOI period relatively accurately and may serve as a noninvasive, reliable and same cycle test for ER in reproductive clinics.

**Trial registration:**

Chinese Clinical Trial Registry: ChiCTR-DDD-17013375. Registered 14 November 2017, http://www.chictr.org.cn/index.aspx.

**Supplementary Information:**

The online version contains supplementary material available at 10.1186/s12958-023-01070-0.

## Background

An ideal synchrony between the embryo and the receptive endometrium is necessary for successful implantation. The receptive period of the endometrium, referred to as the window of implantation (WOI), normally occurs on the 19th to 24th day of a 28-day cycle. Previous studies have demonstrated that the pregnancy rate would significantly reduce when implantation is not performed during the WOI [1, 2]. However, the optimal WOI lasts for less than 48 hours and varies widely between individuals [3]. Abnormal endometrium receptivity (ER), including WOI shifts and pathologic injury, has been observed in numerous patients with repeated implantation failure (RIF) [4–6]. Therefore, an approach for evaluating ER status is urgently needed, especially in the field of assisted reproductive technology (ART).

To fulfill this requirement, several methods have been proposed in recent decades, such as ultrasound examination [7–9], histologic analysis [10], and morphological markers [11–13]. However, none have proven to be an ideal predictor of endometrial receptivity. With advances in molecular biological technologies, our understanding of the molecular mechanism of embryo implantation has significantly improved. In 2011, a 238-gene endometrial receptivity array (ERA) using an RNA expression microarray was developed by Diaz-Gimeno et al. [14]. The ERA method is capable of identifying different stages of the endometrial cycle, which are known as the pre-receptive (PR), receptive (RE), and post-receptive (PO) stages. Although not independent confirmations, the method’s accuracy and reproducibility have been reported to be reliable in a series of studies [15, 16]. Recent studies have demonstrated that pregnancy outcomes of patients with displaced WOI and infertile couples with conventional IVF can be improved by personalized embryo transfer (pET) guided by the ERA test, even without a history of RIF [17, 18]. In addition, relevant results indicate that transcriptomic and proteomic markers serve as promising tools for ER assessment [19, 20]. Although numerous differentially expressed genes (DEGs) involved in endometrial receptivity have been identified by previous studies, the overlap between these results is rather limited. One explanation might be that sample sizes, individual differences and microarray platforms differ between studies. Next-generation, high-throughput RNA sequencing (RNA-seq) is another powerful tool for comprehensively analyzing the whole transcriptome. RNA-seq is better than a microarray in terms of dynamic range, background noise, and identifying different transcripts [21, 22]. Another limitation for current diagnostic tools of endometrial receptivity is rooted in the need for invasive tissue sampling by endometrial biopsy. The endometrial RNA expression profile could be altered due to the small injuries caused by invasive sampling [23]. In addition, local injury to the endometrium was reported to have a negative impact on implantation [24]; therefore, it is inappropriate to perform endometrial tissue sampling tests and guide implantation in the same active cycle. It is necessary to develop a noninvasive diagnostic tool to accurately predict the WOI.

Uterine fluids are an important medium of communication between the embryo and endometrium and include an admixture of endometrial secretions, plasma transudates, and oviductal fluid [25]. Uterine fluid contains extracellular vesicles, RNAs, DNAs, regulatory proteins, ions, lipids and other bioactive factors and plays an important role in embryo implantation [26]. Thus, the high-throughput sequencing of uterine fluid provides an opportunity to find noninvasive biomarkers of endometrial receptivity for clinical use. The aspiration of uterine fluid prior to embryo transfer does not affect the embryo implantation rate [27], suggesting the feasibility of developing a noninvasive diagnostic tool based on uterine fluid. However, few transcriptional studies have focused on endometrial receptive markers from uterine fluid. A previous study [28] identified 53 candidate genes predictive of endometrial receptivity by using microarray technology to analyze uterine fluid, clinical diagnostic tests have not been conducted.

The aim of our study was to investigate the feasibility of predicting ER with biomarkers from uterine fluid and to establish a noninvasive RNA-seq-based endometrium receptivity test (nirsERT) with the potential to be used in reproductive clinics.

## Materials and methods

### Study design

The main objective of this study was to establish a prediction tool for endometrial receptivity using transcriptome sequencing data and to evaluate the feasibility of a noninvasive endometrial receptivity test using uterine fluid specimens. First, from November 2017 to December 2018, participants were recruited for us to identify differentially expressed genes (DEGs) in pre-receptive, receptive and post-receptive uterine fluid by transcriptome sequencing and expression profile analysis and to build the nirsERT model applying a random forest (RF) machine learning algorithm. To limit interference from confounding variables affecting ER, the inclusion criteria for IVF patients were set as follows: 20–39 years of age; body mass index (BMI) = 18–25 kg/m2; patients with a history of a intrauterine pregnancy/pregnancies who underwent the first IVF cycle due to tubal factors alone or patients who undergoing the first IVF cycle due to male factors alone; a regular menstrual cycle length (25–35 days) with spontaneous ovulation; normal ovarian reserves (baseline FSH < 10 mIU/mL, antimullerian hormone > 1.5 ng/ml, and antral follicle count > 5); able to be followed up to assess the pregnancy outcome; and successful intrauterine pregnancy after the first embryo transfer (ET). Intrauterine pregnancy was defined as the presence of a gestational sac with or without fetal heart activity in the uterine cavity as evaluated by ultrasound 4–5 weeks after ET. To establish the prediction tool, normal ER status was defined as a successful intrauterine pregnancy.

Second, from January to April 2019, participants were recruited to demonstrate the accuracy of the nirsERT in predicting the WOI. The inclusion criteria for patients from which we collected uterine fluid on the day of cryothaw blastocyst transfer were as follows: 20–39 years of age; BMI = 18–25 kg/m^2^; ultrasound showing an endometrial thickness of ≥8 cm and an endogenous serum progesterone level of ≤1.2 ng/ml on the day of progesterone administration/LH peak; and transferred embryos with high-quality blastocysts (blastocysts ≥3 BB on Day 5 and Day 6, graded based on the Gardner system) [29].

The following exclusion criteria were applied: endometrial diseases (including intrauterine adhesions, endometrial polyps, endometritis, endometrial tuberculosis, endometrial hyperplasia, and a thin endometrium); hydrosalpinx without proximal tubal ligation; submucous myomas, intramural hysteromyomas, or adenomyomas protruding toward the uterine cavity; endometriosis (stages III–IV); uterine malformations; and other medical or surgical comorbidities identified by consulting medical records, physical examination, blood tests, B-ultrasound and X-ray examination.

In the validation group, all patients received the nirsERT and were followed up to 4–5 weeks after ET to determine intrauterine pregnancy by ultrasound. Subsequently, all patients diagnosed with an intrauterine pregnancy were followed up until delivery.

### Ethics statement

The present study was conducted at the Center for Reproductive Medicine at Xiangya Hospital of Central South University with permission from the Ethics Committee of Reproductive Medicine. This study is registered with the Chinese Clinical Trial Registry (No. ChiCTR-DDD-17013375).

### Uterine fluid collection, processing and transcriptome sequencing

All patients provided written informed consent before sample collection. For patients included in model construction, uterine fluid samples were collected at three time points in the natural cycle preceding the first IVF cycle. Ultrasound was initiated from day 10 of the menstrual cycle to monitor ovulation. Blood LH levels were dynamically measured daily when the follicle diameter was > 14 mm. Patients continued to undergo daily ultrasound monitoring of ovulation until follicular discharge. Uterine fluid was collected using an embryo transfer catheter (Cook Medical; America) on days 5, 7, and 9 (LH + 5, LH + 7, and LH + 9, respectively) after the LH surge (denoted as LH + 0). For patients in the model validation group, uterine fluid was collected on the day of blastocyst transfer before embryo transfer. (Transfers of frozen-thawed blastocysts were performed 7 days after the LH surge of the natural cycle/5 days after the progesterone supplementation of hormone replacement (HRT) cycles).

Sampling was performed as follows. The cervix was cleansed with saline before sampling. After the outer catheter of the embryo transfer catheter was inserted through the cervix to a depth of 4 cm from the external cervical os, the inner catheter was introduced into the uterine cavity to a point 1–2 cm from the uterine fundus to avoid contamination with cervical mucus. A 2.5 mL syringe was connected to the inner catheter, and suction was applied. The inner catheter was withdrawn within the external catheter before the external catheter was withdrawn from the uterus. Approximately 5–10 μL of uterine fluid obtained was immediately placed into 20 μL of RNA-later buffer (AM7020; Thermo Fisher Scientific, Waltham, MA, USA) for RNA stabilization, sealed, and cryopreserved at − 20 °C. Sequencing analysis was carried out within 7 days after sampling.

Total RNA was extracted by using an RNeasy Micro Kit (74,004; Qiagen, Hilden, Germany) according to the manufacturer’s instructions. Quality control of RNA was performed with a Qubit HS RNA Kit (Q32855, Thermo Fisher Scientific, Waltham, MA, USA) and Agilent Bioanalyzer 2100 (G2939BA, Agilent Technologies, Santa Clara, CA, USA). Reverse transcription and library preparation were conducted using the MALBAC® Platinum single-cell RNA amplification kit and Transposon Library Prep Kit (KT110700796 and XY045, Yikon Genomics, Suzhou, China). Qualified libraries were sequenced using the Illumina HiSeq 2500 platform with a single-end read length of 140 bp. An average number of 5 million reads was generated for each library. Low-quality bases and adapters were filtered or trimmed by the Trimmomatic tool (version 0.33). Filtered reads were then mapped to the human reference genome (ensembl primary assembly, version GRCh37) using STAR [30]. The RNA expression level was normalized based on FPKM (fragments per kilobase million) of each gene by RNA-SeQC (version 1.1.8) [31]. Base-2 logarithmic transformation of FPKM was conducted for further analyses.

### Detection of differentially expressed genes

Differentially expressed genes (DEGs) among different endometrial receptivity conditions were identified by analysis of variance (ANOVA). The equation is written as follows:

$${Y}_{gi j}={\mu}_g+{T}_{gi}+{S}_{gj}+{\varepsilon}_{gi j}$$

where *μ*_*g*_ represents the mean expression level of gene g; *T*_*gi*_ is the gene-specific treatment effect referring to the status of a natural cycle or hormone replacement therapy when uterine fluid was obtained, ${T}_{gi}\sim \left(0,{\sigma}_{T_g}^2\right)$; *S*_*gj*_ is the gene-specific endometrial receptivity stage effect with three levels (pre-receptivity, receptivity, and post-receptivity), ${S}_{gj}\sim \left(0,{\sigma}_{S_g}^2\right)$; and *ε*_*gij*_ is the gene-dependent residual error, ${\varepsilon}_{gij}\sim \left(0,{\sigma}_{\varepsilon_g}^2\right)$. An F-test was applied to statistically assess the equality of variances between *S*_*j*_ and *ε*_*ijk*_ for each gene, showing whether each gene was differentially expressed among different endometrial receptivity stages. Because RNA-Seq analysis involves multiple statistical tests, the false discovery rate (FDR) was used to adjust the *p*-value (q-value) for statistical inference. A Gene Ontology (GO) annotation and functional analysis of these DEGs was conducted with the DAVID tool [32].

### Co-expression network construction and visualization

Co-expression modules in the endometrial receptivity process were detected by weighted gene co-expression network analysis (WGCAN) [33]. Applying WGCNA, we then identified key modules significantly correlated with endometrial receptivity stages. Cytoscape software (version 3.8.1) was then used to visualize the interaction networks with different co-expression key modules [34].

### Biomarker identification and performance validation

To identify biomarkers for predictive model construction, a post hoc Tukey HSD (honestly significant difference) test from an ANOVA was applied for pairwise comparisons of three receptive levels. Genes showing significant differences in all pairwise tests were detected to maximally distinguish each receptive stage. Expression values of these biomarkers were then inputted as features for the machine learning method-random forest to train the pattern on three ER conditions (pre-receptivity, receptivity, and post-receptivity). The most important features (gene expression) were further selected by R package random forest based on two measures (mean decrease accuracy and mean decrease gini). Mean accuracy, sensitivity, specificity, the positive predictive value and the negative predictive valuewere determined through 10-fold cross-validation.

### Statistical analysis

Continuous data subject to a normal distribution were expressed as the mean ± SD and were compared using independent-samples t-tests. Continuous data subject to a skewed distribution were expressed as the median and interquartile range (IQR) and were compared using an independent-samples Mann-Whitney U test. Categorical data were expressed as counts and percentages and were determined to be statistically significant using a chi-square test or Fisher’s exact test. A two-sided *P*-value equal to or less than 0.05 was considered to be statistically significant. Statistical analysis was performed using IBM SPSS software (Version 23.0, IBM Corp.)

## Results

### Participants

**Fig. 1:**
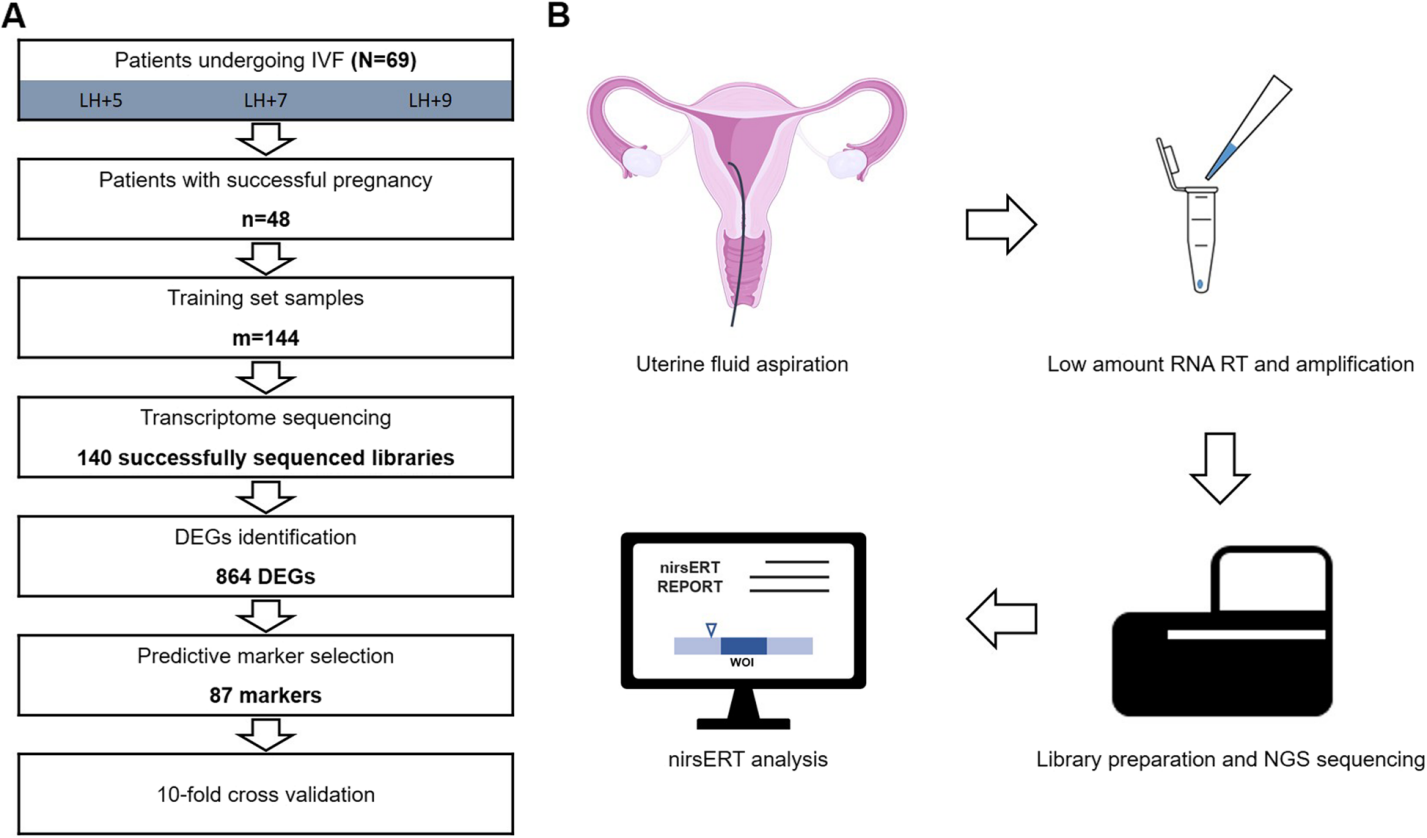
Flow diagram of the establishment and application of the noninvasive RNA-seq-based endometrial receptivity test

To establish the nirsERT model, we collected uterine fluid of three different receptive stages (pre-receptive, receptive and post-receptive) in the same cycle from IVF patients with normal WOI timing for RNA-seq. Sixty-nine participants were recruited, 21 patients who were not pregnant in the first embryo transfer cycle after the sampling cycle were excluded, and 48 patients with successful intrauterine pregnancies were studied to build the nirsERT model (Fig. 1). Baseline clinical characteristics are shown in supplementary Table S1.

### Uterine fluid RNA extraction and sequencing

To perform transcriptome sequencing, we collected 144 uterine fluid specimens from 48 participants and extracted total RNA using a commercial kit. As expected, the yield of RNA was relatively low, ranging from 0 to 1160 ng with an average of 148 ng. Almost one-third of RNA samples were below the detection limit of the Qubit RNA HS assay kit (0.25 ng/μL). Normally, it is difficult to construct sequencing libraries with less than 1 ng of total RNA. To address this issue, we utilized a commercial kit for reverse transcription and amplification with a low amount of RNA.

We first validated the repeatability of transcriptome sequencing combined with the above mentioned kit (see supplementary methods). The Spearman correlation between different initial amounts of RNA was above 0.95, demonstrating the stability and repeatability of this method with at least 0.2 ng RNA (Supplementary Fig. S1). Then, we processed the 144 RNA samples according to the same protocol. As a result, 140 NGS libraries were successfully constructed and sequenced, generating an average of 5.5 million raw reads per library. All sequencing data of this study have been deposited into CNGB Sequence Archive (CNSA) of China National GeneBank DataBase (CNGBdb, https://db.cngb.org/) with accession number CNP0002025. A total of 632 million high-quality reads, representing approximately 82.1% of raw data, were mapped to the human reference genome (ensembl primary assembly, version GRCh37). The number of mapped genes ranged from 9591 to 17,913 in each library.

### DEGs detection and functional analysis

**Fig. 2:**
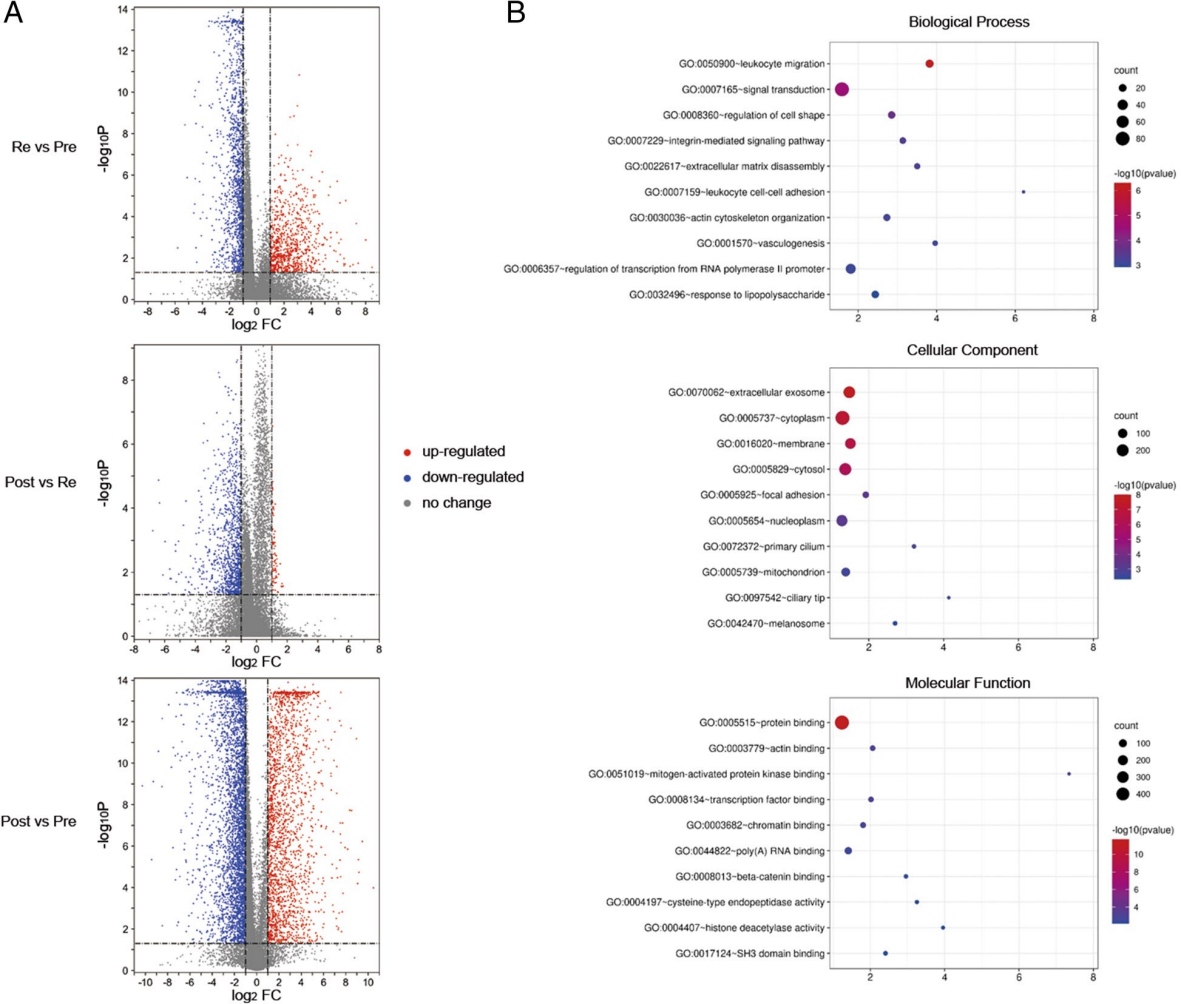
Differential expression analysis and functional enrichment across endometrial receptivity conditions. **A** Volcano plot for RNA profile of PR, PO and RE samples. The cut-off is set as 0.05 for *p*-value and 2 for fold-change. **B** Bubble chart for functional enrichment analysis of DEGs. X-axis: fold of enrichment. Color of bubble: log_10_ (*p*-value). Size of bubble: number of genes enriched in the corresponding GO term

To identify differentially expressed genes (DEGs) among the pre-receptivity, receptivity, and post-receptivity stages, an ANOVA (analysis of variance) was applied to process the log2 transformed transcriptomic data. As a result, 864 DEGs were detected across the three different ER statuses. Notably, there were relatively more downregulated DEGs between the post-receptivity and receptivity stages (Fig. 2A). An unsupervised hierarchical clustering of the DEGs revealed three distinct groups. An GO analysis of these DEGs was conducted with the DAVID tool [32]. The DEGs were significantly enriched in 71 biological process (BP) terms, 38 cellular component (CC) terms and 25 molecular function (MF) terms. The top 1 enriched terms for each category were identified as signal transduction (GO:0007165), cytoplasm (GO:0005737), and protein binding (GO:0005515) (Table 1 and Fig. 2B).

**Table 1:** GO enrichment analysis of DEGs from uterine fluid samples

Category	Term	Gene count	***p***-value	Fold Enrichment	FDR
**Biological Process**	GO:0007165 ~ signal transduction	83	2.92E-05	1.59	0.05
	GO:0045944 ~ positive regulation of transcription from RNA polymerase II promoter	60	1.40E-02	1.36	22.49
	GO:0000122 ~ negative regulation of transcription from RNA polymerase II promoter	50	2.60E-03	1.54	4.61
	GO:0045893 ~ positive regulation of transcription, DNA-templated	39	1.98E-03	1.68	3.53
	GO:0006357 ~ regulation of transcription from RNA polymerase II promoter	36	8.56E-04	1.81	1.54
	GO:0006954 ~ inflammatory response	26	3.67E-02	1.52	49.24
	GO:0043065 ~ positive regulation of apoptotic process	24	9.19E-03	1.77	15.42
	GO:0050900 ~ leukocyte migration	21	5.05E-07	3.82	0.00
	GO:0001525 ~ angiogenesis	21	2.68E-03	2.09	4.75
	GO:0008360 ~ regulation of cell shape	18	1.87E-04	2.85	0.34
**Cellular Component**	GO:0005737 ~ cytoplasm	298	7.41E-08	1.30	0.00
	GO:0005634 ~ nucleus	270	8.01E-03	1.13	10.94
	GO:0005829 ~ cytosol	200	8.78E-07	1.37	0.00
	GO:0070062 ~ extracellular exosome	183	1.55E-08	1.48	0.00
	GO:0005654 ~ nucleoplasm	157	5.89E-04	1.28	0.85
	GO:0016020 ~ membrane	146	2.40E-07	1.51	0.00
	GO:0005739 ~ mitochondrion	81	2.41E-03	1.38	3.42
	GO:0005615 ~ extracellular space	75	2.61E-02	1.27	31.66
	GO:0048471 ~ perinuclear region of cytoplasm	38	3.75E-02	1.39	42.34
	GO:0009986 ~ cell surface	34	3.70E-02	1.43	41.92
**Molecular Function**	GO:0005515 ~ protein binding	492	2.66E-12	1.23	0.00
	GO:0044822 ~ poly(A) RNA binding	72	3.03E-03	1.41	4.64
	GO:0008270 ~ zinc ion binding	66	4.84E-02	1.24	54.05
	GO:0042803 ~ protein homodimerization activity	46	2.32E-02	1.39	30.79
	GO:0043565 ~ sequence-specific DNA binding	33	4.91E-02	1.40	54.63
	GO:0003682 ~ chromatin binding	32	1.88E-03	1.80	2.90
	GO:0005102 ~ receptor binding	27	1.07E-02	1.69	15.49
	GO:0003779 ~ actin binding	26	9.10E-04	2.06	1.42
	GO:0008134 ~ transcription factor binding	26	1.23E-03	2.02	1.90
	GO:0044212 ~ transcription regulatory region DNA binding	17	3.33E-02	1.76	41.17

To further investigate the functional module of DEGs in the uterine fluid samples, we used the weighted gene coexpression network analysis (WGCNA) algorithm to analyze transcription regulatory networks. As a result, 4 coexpression network modules, 3 of which were MEturquoise, MEyellow and MEblue modules, were found to be highly significantly correlated with the ER stage. Four hub genes, ECI2 (MEturquoise), ATP6V1B2 (MEyellow), CXCL16 (MEblue) and SELP (MEgray), were then identified based on the highest level of intramodular connectivity found in the four coexpression modules (Table 2). The MEturquoise module included most DEGs, representing 59.1% (511/864) of the DEGs. The analysis also shows the strongest correlation with the ER stage with a correlation value of − 0.7. A functional enrichment analysis shows that genes in the MEturquoise module are involved in transcription regulation, such as epigenic modification-related pathways. MEblue genes are enriched in GTPase-mediated signal transduction, while MEyellow genes play roles in biomacromolecule transport and cell-cell adherens junctions. This result reflects the overall involvement of DEGs detected in the uterine fluid in endometrium-embryo crosstalk related biological processes, which include cell-cell communication, signal reception and transduction, and a series of cellular responses including the transcription and translation of proteins responsible for embryo implantation.

**Table 2:** WGCNA analysis of DEGs from uterine fluid

Module	Number of genes	Hub gene	Module-receptivity relationships	DAVID cluster	*p-value	Enrichment score
ME turquoise	510	ECI2	−0.7	GO:0016575 ~ histone deacetylation	0.0479	3.44
				GO:0004407 ~ histone deacetylase activity	0.0371	
				GO:0016581 ~ NuRD complex	0.0416	
ME blue	265	CXCL16	0.55	GO:0051056 ~ regulation of small GTPase mediated signal transduction	0.0192	3.5
				GO:0043547 ~ positive regulation of GTPase activity	0.0385	
				GO:0005096 ~ GTPase activator activity	0.0557	
ME yellow	78	ATP6V1B2	0.69	GO:0042470 ~ melanosome	0.0133	2.4
				GO:0045121 ~ membrane raft	0.0935	
				GO:0005913 ~ cell-cell adherens junction	0.0935	

*: Benjamini adjusted *p*-value

### Establishing and validating the ER predictive tool

**Fig. 3:**
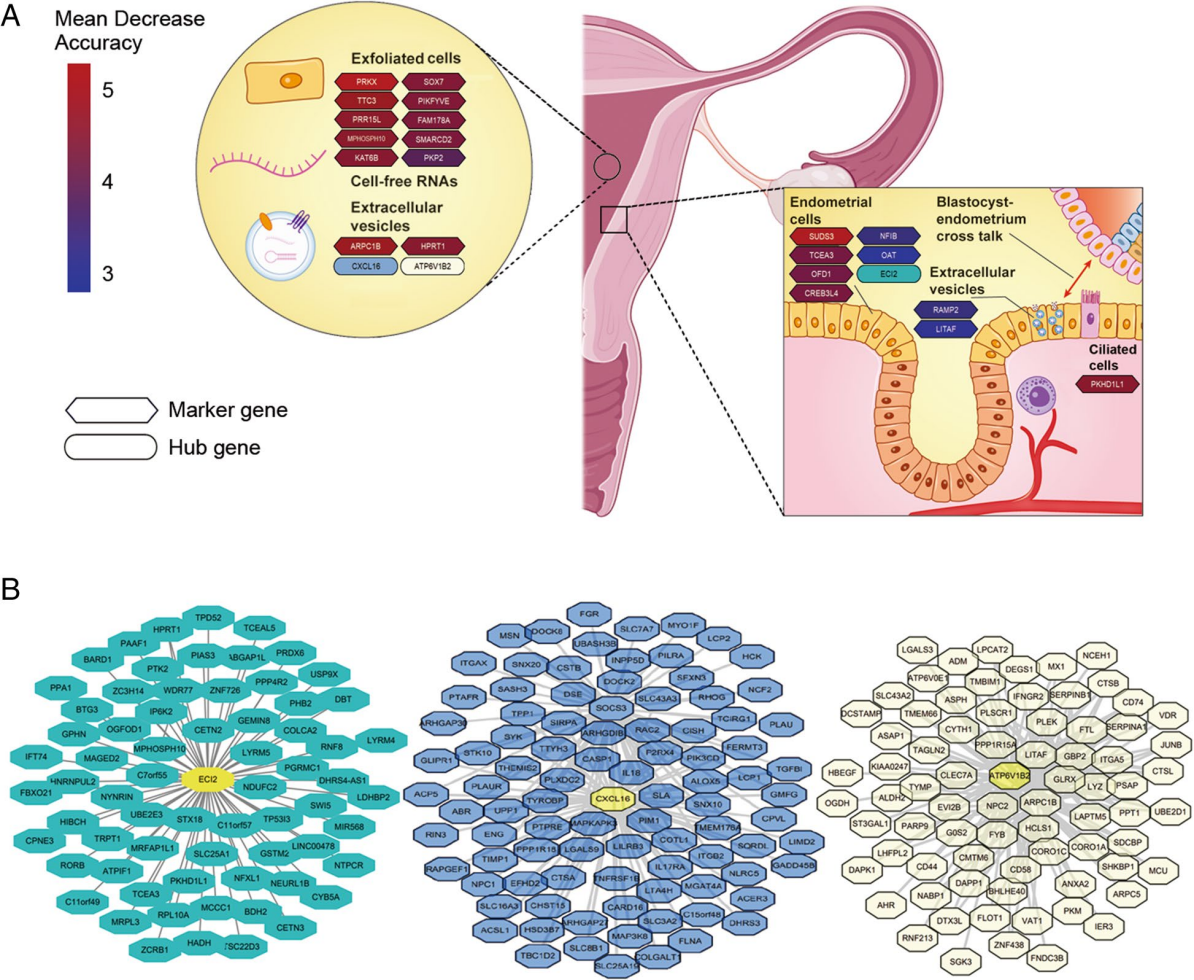
Partial predictive markers of the nirsERT. **A** Inferred source of marker and hub genes for the nirsERT; **B** Co-expression modules of uterine fluid DEGs generated via WGCNA

**Fig. 4:**
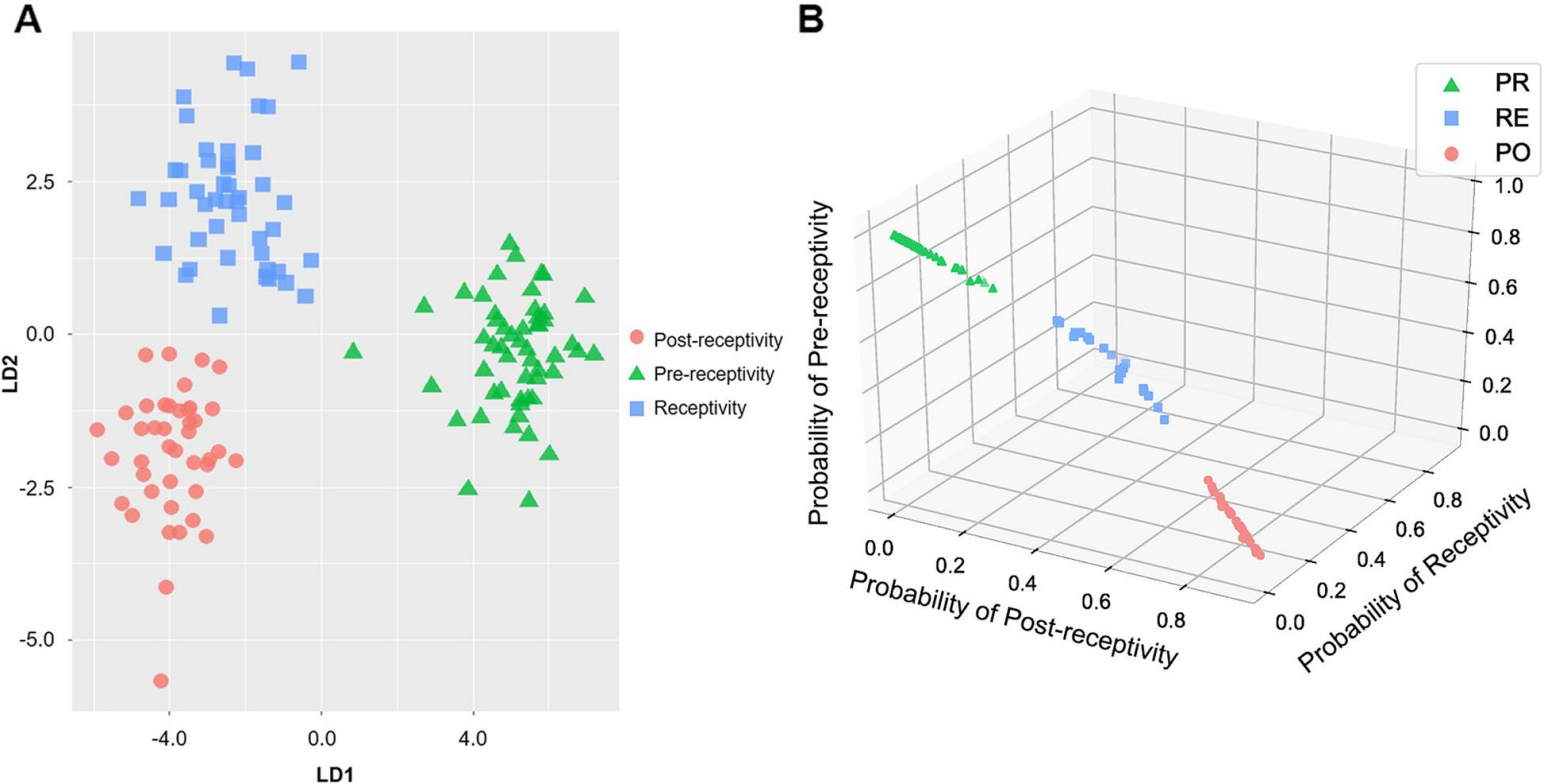
Establishment and validation of the nirsERT. **A** Clustering the training set via LDA using selected predictive markers; **B** Prediction results of training set samples with a probability threshold of 0.6. Pink dot: samples predicted as post-receptivity. Green triangle: samples predicted as pre-receptivity. Blue square: samples predicted as receptivity

With Tukey’s test from an ANOVA, we selected genes with varying expression identified from each pairwise comparison of receptive stages (pre-receptivity versus receptivity, receptivity versus post-receptivity, and pre-receptivity versus post-receptivity). We therefore applied the expression pattern of these DEGs as training features for ER status classification using the random forest method. A random forest-based feature importance analysis with model prediction based on mean decrease accuracy and the Gini index was performed [35], resulting in 87 predictive markers (Table 3). To improve the power of the predictive tool, we included three hub genes as additional markers (Fig. 3), resulting in an nirsERT. Linear discriminant analysis (LDA) shows that the three ER conditions (pre-receptivity, receptivity, and post-receptivity) were distinctly classified by the expression patterns of these transcriptomic markers (Fig. 4A). To assess the performance of the present predictor, 10-fold cross-validation were applied. We obtained a mean accuracy of 93.0%, a mean specificity of 95.9%, and a mean sensitivity of 90.0%. Uterine fluid samples of different ER conditions could be well separated by setting a probability threshold of 0.6 (Fig. 4B).

**Table 3:** List of predictive markers selected by random forest algorithm

HGNC ID	Approved symbol	Approved name	Mean Decrease Accuracy
HGNC:9441	PRKX	protein kinase X-linked	5.21
HGNC:8910	PGR	progesterone receptor	5.05
HGNC:29545	SUDS3	SDS3 homolog, SIN3A corepressor complex component	4.95
HGNC:704	ARPC1B	actin related protein 2/3 complex subunit 1B	4.72
HGNC:12393	TTC3	tetratricopeptide repeat domain 3	4.69
HGNC:28149	PRR15L	proline rich 15 like	4.54
HGNC:7213	MPHOSPH10	M-phase phosphoprotein 10	4.53
HGNC:20313	PKHD1L1	PKHD1 like 1	4.51
HGNC:5157	HPRT1	hypoxanthine phosphoribosyltransferase 1	4.48
HGNC:17582	KAT6B	lysine acetyltransferase 6B	4.48
HGNC:18196	SOX7	SRY-box transcription factor 7	4.41
HGNC:23785	PIKFYVE	phosphoinositide kinase, FYVE-type zinc finger containing	4.4
HGNC:17814	SLF2	SMC5-SMC6 complex localization factor 2	4.36
HGNC:11107	SMARCD2	SWI/SNF related, matrix associated, actin dependent regulator of chromatin, subfamily d, member 2	4.35
HGNC:4461	GPM6B	glycoprotein M6B	4.33
HGNC:2470	CSRP2	cysteine and glycine rich protein 2	4.31
HGNC:18854	CREB3L4	cAMP responsive element binding protein 3 like 4	4.31
HGNC:11615	TCEA3	transcription elongation factor A3	4.28
HGNC:17947	THEM4	thioesterase superfamily member 4	4.26
HGNC:2567	OFD1	OFD1 centriole and centriolar satellite protein	4.25
HGNC:4330	GLRX	glutaredoxin	4.24
HGNC:24663	RABGAP1L	RAB GTPase activating protein 1 like	4.2
HGNC:17811	AMOTL1	angiomotin like 1	4.19
HGNC:4183	GBP2	guanylate binding protein 2	4.14
HGNC:26323	ANKRD35	ankyrin repeat domain 35	4.13
HGNC:14651	PPIH	peptidylprolyl isomerase H	4.11
HGNC:16462	STRBP	spermatid perinuclear RNA binding protein	4.08
HGNC:17717	STK39	serine/threonine kinase 39	4.05
HGNC:25585	OGFOD1	2-oxoglutarate and iron dependent oxygenase domain containing 1	4.04
HGNC:7784	NFIA	nuclear factor I A	4.02
HGNC:20340	PRICKLE2	prickle planar cell polarity protein 2	4
HGNC:9024	PKP2	plakophilin 2	3.99
HGNC:21923	STEAP4	STEAP4 metalloreductase	3.94
HGNC:4171	GATA2	GATA binding protein 2	3.93
HGNC:21150	RNF125	ring finger protein 125	3.89
HGNC:6846	MAP2K6	mitogen-activated protein kinase kinase 6	3.85
HGNC:411	ALDH3B2	aldehyde dehydrogenase 3 family member B2	3.85
HGNC:19300	STX19	syntaxin 19	3.83
HGNC:4881	HEY2	hes related family bHLH transcription factor with YRPW motif 2	3.83
HGNC:18296	PPP4R2	protein phosphatase 4 regulatory subunit 2	3.82
HGNC:5464	IGF1	insulin like growth factor 1	3.81
HGNC:28990	ZNF516	zinc finger protein 516	3.8
HGNC:25569	NKAPD1	NKAP domain containing 1	3.78
HGNC:10524	SALL1	spalt like transcription factor 1	3.76
HGNC:25764	RMI1	RecQ mediated genome instability 1	3.75
HGNC:17925	TFCP2L1	transcription factor CP2 like 1	3.74
HGNC:20814	ZNF436	zinc finger protein 436	3.74
HGNC:30447	PLD6	phospholipase D family member 6	3.74
HGNC:253	ADH5	alcohol dehydrogenase 5 (class III), chi polypeptide	3.72
HGNC:24944	DDIT4	DNA damage inducible transcript 4	3.71
HGNC:15513	SMYD3	SET and MYND domain containing 3	3.65
HGNC:29652	WDR77	WD repeat domain 77	3.61
HGNC:22201	TCAF1	TRPM8 channel associated factor 1	3.6
HGNC:8154	OPRK1	opioid receptor kappa 1	3.59
HGNC:8013	HMGN5	high mobility group nucleosome binding domain 5	3.58
HGNC:18856	CREB3L1	cAMP responsive element binding protein 3 like 1	3.57
HGNC:28204	NTPCR	nucleoside-triphosphatase, cancer-related	3.57
HGNC:18122	SOX17	SRY-box transcription factor 17	3.54
HGNC:20150	RAB15	RAB15, member RAS oncogene family	3.52
HGNC:941	BAG5	BAG cochaperone 5	3.5
HGNC:7785	NFIB	nuclear factor I B	3.49
HGNC:9844	RAMP2	receptor activity modifying protein 2	3.48
HGNC:3821	FOXO3	forkhead box O3	3.46
HGNC:8995	PIP5K1B	phosphatidylinositol-4-phosphate 5-kinase type 1 beta	3.39
HGNC:33941	SLC35E2B	solute carrier family 35 member E2B	3.38
HGNC:4908	HIBCH	3-hydroxyisobutyryl-CoA hydrolase	3.36
HGNC:5209	HSD11B2	hydroxysteroid 11-beta dehydrogenase 2	3.35
HGNC:6813	MAGED1	MAGE family member D1	3.34
HGNC:18757	RHOBTB3	Rho related BTB domain containing 3	3.32
HGNC:4253	GGTA1	glycoprotein alpha-galactosyltransferase 1 (inactive)	3.32
HGNC:4254	GGTA2P	glycoprotein alpha-galactosyltransferase 2, pseudogene	3.29
HGNC:19990	ANAPC4	anaphase promoting complex subunit 4	3.24
HGNC:8062	NUP153	nucleoporin 153	3.23
HGNC:12805	XDH	xanthine dehydrogenase	3.23
HGNC:23696	TIPARP	TCDD inducible poly(ADP-ribose) polymerase	3.22
HGNC:19391	SOCS3	suppressor of cytokine signaling 3	3.21
HGNC:29147	ZNF652	zinc finger protein 652	3.2
HGNC:29947	TRAK1	trafficking kinesin protein 1	3.18
HGNC:13071	PATZ1	POZ/BTB and AT hook containing zinc finger 1	3.18
HGNC:1132	BTG3	BTG anti-proliferation factor 3	3.15
HGNC:30747	COPS2	COP9 signalosome subunit 2	3.13
HGNC:7541	MXRA7	matrix remodeling associated 7	3.13
HGNC:4403	GNG11	G protein subunit gamma 11	3.11
HGNC:31412	SWI5	SWI5 homologous recombination repair protein	3.11
HGNC:16841	LITAF	lipopolysaccharide induced TNF factor	3.1
HGNC:7852	NME4	NME/NM23 nucleoside diphosphate kinase 4	3.07
HGNC:7391	MSX1	msh homeobox 1	3.00

### Retrospective observation of a small cohort of patients undergoing IVF

To further evaluate the accuracy of the nirsERT, we analyzed the correlation between the predicted results of the nirsERT and the pregnancy outcomes. Twenty-two participants were recruited and their baseline clinical characteristics are shown in Table 4. Twenty-two uterine fluid samples from IVF patients were collected on the day of blastocyst transfer before embryo transfer and tested. Intrauterine pregnancy was determined by ultrasound 28 days after embryo transfer. The success rate of sequencing was 95.4% (21/22) with 1 library failing to pass the quality control procedure. As a result, 18 patients (85.7%, 18/21) were predicted to have a normal WOI, whereas 3 (14.3%, 3/21) and 0 were predicted to have delayed and advanced WOIs, respectively. The intrauterine pregnancy rate (IPR) was 77.8% (14/18) and live birth rate (LBR) was 72.2% (13/18) for patients with a normal WOI. There was no successful pregnancy in patients with a displaced WOI, significantly differing from those with a normal WOI (*P* < 0.05). The overall IPR and LBR for all patients were recorded as 63.6% (14/22) and 59.1% (13/22), respectively (Table 4).

**Table 4:** Baseline clinical characteristics, nirsERT results and clinical outcomes of 22 patients undergoing IVF

	Normal WOI	Displaced WOI	*P*-value	Detection failed	Total
Date of transfer	LH + 7/P + 5	LH + 7/P + 5		LH + 7/P + 5	/
Predicted result	Receptivity	Pre-receptivity		/	/
No. of patients	18	3		1	22
Age, Mean ± SD, y	30.4 ± 3.87	28.67 ± 3.51	0.466	33	/
BMI, Mean ± SD, kg/m2	20.95 ± 2.49	22.53 ± 0.76	0.297	21.6	/
Infertility duration, Median (IQR), y	2(1–5)	2^a^	0.387	5	/
AMH, Median (IQR), ng/ml	2.90(2.36–5.29)	6.32^a^	0.430	3.10	/
FSH, Mean ± SD, mIU/ml	5.74 ± 0.88	5.70 ± 1.33	0.952	4.67	/
Endometrial thickness, Mean ± SD, mm	11.21 ± 2.03	9.33 ± 2.14	0.158	11.80	/
P levels on the day of progesterone administration/LH peak, Median (IQR), ng/ml	0.90 (0.06–0.20)	0.57^a^	0.164	0.61	/
IVF indication					
Tubal factor (n/%)	14 (77.8%)	1 (33.3%)	0.242	1	/
PCOS (n/%)	3 (16.7%)	2 (66.7%)		/	/
Ovulation disorder (n/%)	2(11.1%)	1 (33.3%)		/	/
Male factor (n/%)	1(5.6%)	0		/	/
Diminished ovarian reserve (n/%)	1(5.6%)	0		/	/
Cycle protocol					
Natural cycle (n/%)	7(38.9%)	1(33.3%)	0.101	/	8
HRT cycle (n/%)	11(61.1%)	2(66.7%)		1	14
No. of intrauterine pregnancy	14	0		0	14
Intrauterine pregnancy rate	77.8%(14/18)	0	0.026	0	63.6%(14/22)
Live birth rate	72.2%(13/18)	0	0.042	0	59.1%(13/22)

*Abbreviations*: *nirsERT* non-invasive RNA-seq-based endometrial receptivity test, *BMI* body mass index, *AMH* antimullerian hormone, *FSH* follicle-stimulating hormone, *PCOS* polycystic ovarian syndrome

^a^Only the Median is listed because there are only three samples

## Discussion

In recent decades, researchers have investigated a variety of means to evaluate the condition of endometrial receptivity. However, limited progress was made until transcriptomic markers were established [28, 36]. Diagnostic tools resulting from the endometrial tissue transcriptome are accurate and reproducible, but their application is hindered by the need for invasive sampling. Thus, developing a noninvasive, precise and reliable method of ERT is a major challenge in reproductive medicine. In this study, a noninvasive ERT method based on RNA-seq was examined for the first time, and we found the following benefits relative to previous studies. (1) RNA-seq can be used to identify more genes and in a more accurate manner than the conventional gene microarray. (2) Rather than sampling over two time points, we collected samples of uterine fluid at three different time points (the pre-receptive, receptive, and post-receptive stages) from the same patient at 48-h intervals during the same cycle. Thus, the study period was shortened, and a highly correlated sample cohort was established, allowing for a more precise analysis of DEGs to identify marker genes for ER. (3) Over 800 DEGs in uterine fluid were analyzed, providing insight into the functions and roles of multiple genes in embryo implantation. It was difficult to perform transcriptome sequencing with uterine fluid samples, as nearly 1/3 of the samples yielded total RNA of less than 0.25 ng/μL. To address this, we utilized a commercial kit designed for single-cell RNA reverse transcription and amplification. The results show high levels of stability and repeatability, and the Spearman correlation between different amounts of total RNA ranging from 0.2 ng to 20 ng was above 0.98. In using this kit, we successfully prepared 140 RNA-seq libraries and constructed the training dataset. However, 4 libraries failed to pass quality control testing, and we assume this might be caused by extremely low amounts of RNA in these uterine fluid samples. To ensure the availability of the nirsERT, it is important to investigate the distribution of the amount of total RNA in the population. In addition, the improvement of uterine fluid aspiration could be helpful in further studies.

According to our previous study [37], 3571 DEGs were identified from endometrial tissue across the ER statuses, and a predictive tool (rsERT) consisting of 175 marker genes was established based on these DEGs. By analyzing integrated data of the two studies, a total of 864 DEGs were identified, including 468 common DEGs shared with the rsERT study, and 396 uterine fluid-specific DEGs. We found that these common DEGs were significantly enriched in extracellular exosome (GO:0070062), cytoplasm (GO:0005737), cytosol (GO:0005829), nucleoplasm (GO:0005654) and protein binding (GO:0005515), supporting the conclusion that RNAs in uterine fluid originate from endometrial tissue cells with exosomes secreted outside of the cell. Unexpectedly, 396 DEGs were specifically observed in uterine fluid samples. These genes are significantly involved in the integrin-mediated signaling pathway (GO:0007229) and in immune responses, such as leukocyte migration (GO:0050900), inflammatory responses (GO:0006954) and responses to lipopolysaccharide (GO:0032496). Uterine fluid may play an independent role in regulating embryo implantation in terms of adhesion and immunity. In addition, approximately 38.2% (330 of 864) of all DEGs were previously reported [13, 14, 38–41], while 61.8% (534 of 864) were first identified to be differentially expressed in all three states of receptivity. Our findings highlight the importance of genes involved in protein binding, signal transduction, and leukocyte migration in uterine fluid. For instance, DEGs enriched in extracellular exosomes (GO:0070062), including SLC25A1 (ENSG00000100075), PLSCR1 (ENSG00000188313), and NME3 (ENSG00000103024), were observed to be significantly related to dynamic changes in sequential receptivity stages, which are assumed to mediate communication between the endometrium and embryo. Other cellular responses and signal transduction-related factors, e.g., RAC2 (ENSG00000128340) and ESR1 (ENSG00000091831), were also observed in our study (see Supplementary Tables S2 and S3).

Four hub genes, ECI2, ATP6V1B2, CXCL16 and SELP were identified via WGCNA. ECI2 encodes a key mitochondrial enzyme involved in the beta-oxidation of unsaturated fatty acids, which may provide energy necessary for the embryo implantation course. The presence of SELP implies the possible mechanism of P-selectin-mediated cell adhesion involved in endometrium-embryo interactions. CXCL16 and its receptor CXCR6 have been reported to play a role in decidualization during pregnancy [42]. ATP6V1B2 (ATPase H+ Transporting V1 Subunit B2) is a transmembrane transporter that may be responsible for transporting biomacromolecule-like secretory proteins to their target locations, such as the extracellular matrix. It is evident that these hub genes may play an important role in endometrium-embryo talk and embryo implantation. Hub genes ECI2, ATP6V1B2, and CXCL16 in three of the coexpression modules, MEturquoise, MEyellow, and MEblue, which are highly correlated with ER, were used as marker genes of ER to build the nirsERT model, increasing the predictive efficacy of the model.

nirsERT consisting of 87 markers and 3 hub genes was selected using a random forest algorithm among 864 DEGs. We compared two predictive tools, the nirsERT and rsERT, established using endometrial tissue samples in our previous study, and only 22 markers were shared for both uterine fluid and tissue samples (Supplementary Table S4). According to the Human Protein Atlas, proteins generated by these genes are located in a variety of subcellular locations [26], such as vesicles (BAG5, RAMP2), the nucleus or nucleoplasm (ZNF652, TRAK1), the cytosol (MAP2K6, RNF125) and cell junctions (PKP2). In addition, a strong correlation for the expression patterns of these genes was observed between uterine fluid and endometrial tissue samples (Supplementary Fig. S2). These results indicate that the source of the common markers could be exfoliated endometrial cells or extracellular vesicles. The performance of the nirsERT with rsERT was also examined using the same standard. 10-fold cross-validation revealed comparable mean accuracy (93.0% vs. 98.4%), mean specificity (95.9% vs. 98.9%) and mean sensitivity levels (90% vs. 97.8%).

We also investigated the markers selected in previous studies [14, 28, 37], and few commonalities were observed (Supplementary Fig. S3). No common marker was selected in all three studies. This may be related to the great differences in RNA expression profiles due to different samples from different populations, different RNA profiling technologies and marker gene screening methods used in each study. However, there is no universal standard for selecting marker genes for endometrial receptivity, and the mechanism of uterine transcriptomic changes involved during the process of embryo implantation is still unknown. Further investigations are required to improve the power and reproducibility of endometrial receptivity prediction.

To verify the accuracy of the nirsERT in predicting endometrial receptivity, uterine fluid collected on the day of blastocyst transfer was subjected to the nirsERT. The accuracy of nirsERT prediction was evaluated by analyzing the correlation between the predicted results and subsequent pregnancy outcomes. The results show that 77.8% (14/18) of patients predicted with a normal WOI had successful intrauterine pregnancies, of which 72.2% (13/18) had live birth, while none of the 3 patients with a displaced WOI had successful pregnancies. It is suggested that the failure of embryo implantation in patients with a displaced WOI may be the result of embryo-endometrial asynchrony. Although four unsuccessful intrauterine pregnancies in patients with a normal WOI were predicted by the nirsERT, 77.8% of IRP is consistent with the view that endometrial factors are responsible for approximately two-thirds of embryo implantation [43, 44]. Therefore, the results also further clinically validate the reliability of the nirsERT in predicting the WOI. Personalized embryo transfer (pET) guided by an nirsERT may contribute to restoring the synchronicity of embryonic and endometrial development, which promotes successful embryo implantation. In addition, the clinical pregnancy rate of routine blastocyst transplantation in our reproductive center reached 55–60%, while the overall intrauterine pregnancy rate of patients with aspiration of uterine fluid on the day of embryo transfer reached 63.6%, suggesting that the aspiration of uterine fluid did not affect embryo implantation. This is similar to another study conducted by our team at the same time, which showed the clinical pregnancy rate and implantation rate were higher in the endometrial fluid aspiration group (62.3% versus 50.8%; 57.1% versus 40.8%, respectively) [45]. The results of this study also indicate the feasibility of uterine fluid aspiration as a non-invasive method. Thus, the nirsERT based on RNA-seq of uterine fluid has the potential to detect and guide pET in the same active cycle, contributing to successful embryo implantation.

It follows that our method currently serves as the most promising approach for ideal pET. However, there are some issues has to confront. Firstly, the nirsERT is not applicable for clinical application in current. There are several aspects we need to improve in the further studies, such as detection period, accuracy, convenience, etc. We aim to provide a rapid turnaround for embryo transfer in a same cycle, by developing techniques like RT-qPCR. In fact, if we could identify several crucial RNA markers for endometrial receptivity, RT-qPCR might be promising. We are planning such studies in the near future. We believe this work is hard but well worth the effort. Secondly, the sample size of this validation study was small, and whether an nirsERT can improve the pregnancy outcomes of IVF patients by guiding pET is not yet known. We think it would be better to design a randomized clinical trial in the future to verify the clinical application value of the nirsERT. Lastly, the mechanism of endometrial receptivity marker genes also needs further investigation to provide a theoretical basis for clinical treatment strategies.

## Conclusions

In summary, we established a noninvasive RNA-seq-based endometrial receptivity test (nirsERT) by a transcriptome sequencing analysis of uterine fluid combined with a random forest algorithm. Endometrial receptive DEGs in uterine fluid may be derived from endometrial tissue cells and have an independent role in embryo implantation. The nirsERT can predict the WOI period relatively accurately and may serve as a noninvasive, reliable and same cycle test for ER in reproductive clinics.

## Supplementary Information

**Additional file 1: **Supplementary Materials and Methods. **Fig. S1.** Analysis of stability and repeatability for the transcriptome sequencing with low amount of RNA. **Fig. S2.** Expression pattern of 22 common markers between nirsERT and rsERT. **Fig. S3.** Venn diagram of predictive markers selected from three independent studies of endometrial receptivity.

**Additional file 2: Table S1.** Clinical characteristics of the participants.

**Additional file 3: Table S2.** Functional enrichment of uterine fluid DEGs.

**Additional file 4: Table S3.** GO enrichment analysis of common and specific DEGs from uterine fluid samples.

**Additional file 5: Table S4.** Functional annotation and Subcellular Location of common markers for nirsERT and rsERT.

## Data Availability

The datasets used and analyzed during the current study are available from the corresponding author on reasonable request.

## Abbreviations

ER: endometrial receptivity
WOI: window of implantation
RNA-Seq: RNA sequencing
nirsERT: a noninvasive RNA-Seq-based endometrial receptivity test
pET: personalized embryo transfer
RIF: repeated implantation failure
ART: assisted reproductive technology
IVF: in vitro fertilization
IVF-ET: in vitro fertilization and embryo transfer
DEGs: differentially expressed genes
BMI: body mass index
ERA: endometrial receptivity array
FSH: follicle-stimulating hormone
LH: luteinizing hormone
