# Supplementary material for: Can biomarkers identified from the uterine fluid transcriptome be used to establish a noninvasive endometrial receptivity prediction tool? A proof-of-concept study
Source: Reprod Biol Endocrinol. 2023 Feb 18;21:20. doi: 10.1186/s12958-023-01070-0 (PMC9938621; doi:10.1186/s12958-023-01070-0)
Supplement: Supplementary file 1 — Additional file 1: Supplementary Materials and Methods. Fig. S1. Analysis of stability and repeatability for the transcriptome sequencing with low amount of RNA. Fig. S2. Expression pattern of 22 common markers between nirsERT and rsERT. Fig. S3. Venn diagram of predictive markers selected from three independent studies of endometrial receptivity. [file 12958_2023_1070_MOESM1_ESM.docx]

**Supplementary**

Can biomarkers identified from the uterine fluid transcriptome be used to establish a noninvasive endometrial receptivity prediction tool? A proof-of-concept study.

Aihua He ^1, 2,3†^, Hong Wu^4†^, Yangyun Zou^5^, Cheng Wan^5^, Jing Zhao^1, 2^, Qiong Zhang^1, 2^, Nenghui Liu^1, 2^, Donge Liu^1, 2^, Yumei Li^1, 2^, Jing Fu^1, 2^, Hui Li^1, 2^, Xi Huang^1, 2^, Tianli Yang^1, 2^, Chunxu Hu^5^, Zhaojuan Hou^1, 2^, Yue Sun^5^, Xin Dong^5^, Jian Wu^5^, Sijia Lu^5^*, Yanping Li^1, 2^*

^1^ Department of Reproductive Medicine, Xiangya Hospital, Central South University, Changsha, Hunan, 410000, China. ^2^ Clinical Research Center for Women’s Reproductive Health in Hunan Province, Changsha, Hunan, 410000, China. ^3^ Department of Reproductive Medicine Center, The Third Xiangya Hospital, Central South University, Changsha,Hunan, 410013, China. ^4^ Department of ENT, Xiangya Hospital, Central South University, Changsha , Hunan, 410000, China. ^5^ Department of Clinical Research, Yikon Genomics Company, Ltd., Suzhou, Jiangsu, 215123, China.

*Correspondence address: Yanping Li: Department of Reproductive Medicine, Xiangya Hospital, 87 Xiangya Road, Changsha City, Hunan Province, 410000, China. E-mail: [liyanp@csu.edu.cn](mailto:liyanp@csu.edu.cn). Sijia Lu: Department of Clinical Research, Yikon Genomics Company, Ltd., #301, Building A3, No. 218, Xinghu Street. Suzhou, Jiangsu, 215123, China. E-mail: lusijia@yikongenomics.com.

† Aihua He and Hong Wu contributed equally to this work and should be considered as co-ﬁrst authors.

**Materials and Methods**

**Validation of transcriptome sequencing with low amount of RNA**

To address the difficulty of constructing sequencing libraries starting with less than 1ng of total RNA, we utilized the MALBAC^®^ Platinum single cell RNA amplification kit (KT110700796, Yikon Genomics, Suzhou, China) for reverse transcription and amplification with low amount of RNA. To ensure the stability and repeatability of this kit, we first performed a pilot study with different amount of total RNA.

Total RNA was extracted from uterine fluid samples by using the RNeasy Micro Kit (74004; Qiagen, city, state, country) according to the manufacturer's instruction. Quality control of RNA was performed with Qubit HS RNA Kit (Q32855; Invitrogen) and Agilent Bioanalyzer 2100 (Agilent Technologies, city, state, country). Then, a set of RNA extracted from three sequential uterine fluid samples was selected, each with the yield over 200ng. We obtained RNA with the amount of 0.02ng, 0.2ng, 2ng, 20ng and 100ng by gradient dilution with RNase-free H_2_O. At last, these diluted RNA was processed with commercial kits to construct sequencing libraries and sequenced with the Illumina HiSeq 2500 platform. An average number of 5 million reads was generated for each library. Fragments per Kilobase Million (FPKM) was calculated by Cufflinks (*1*), the Spearman correlation was then calculated to compare the differences between different initial amounts of RNA.

**Figures**

**
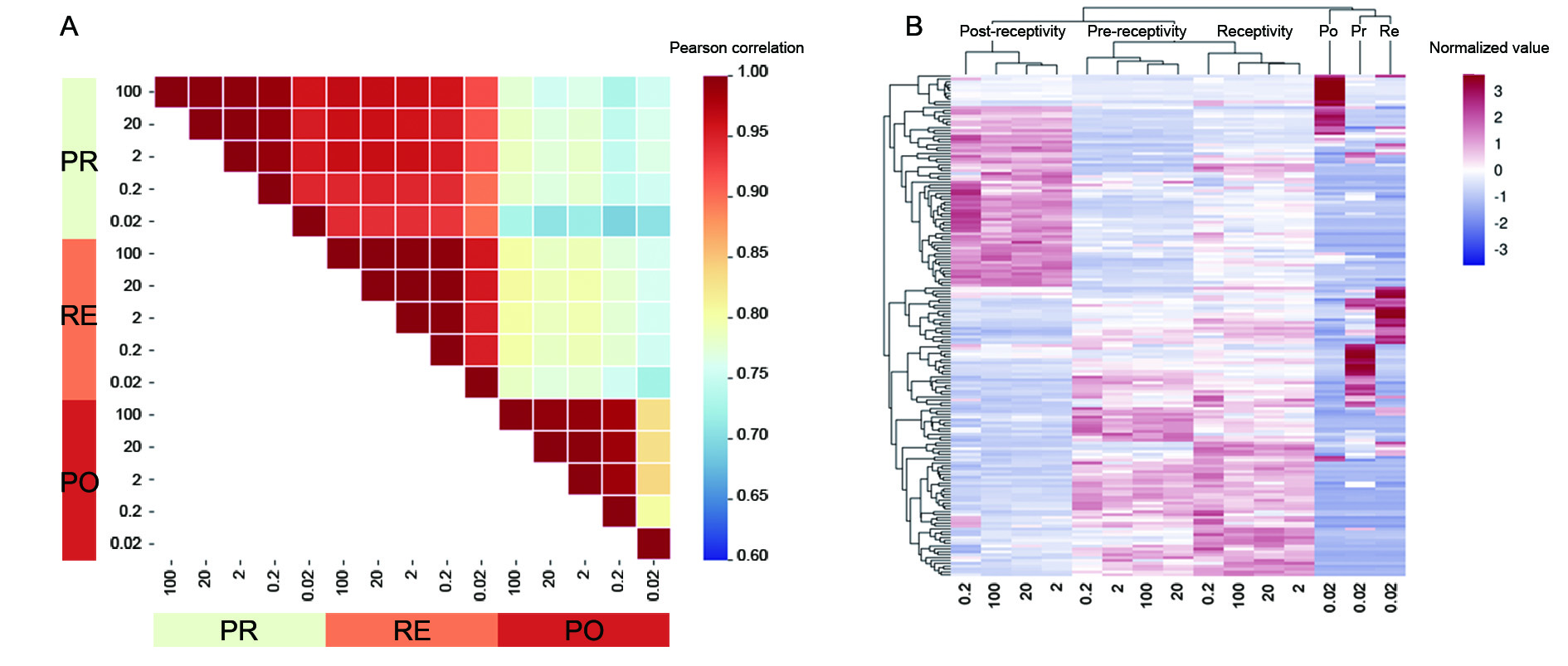
**

**Figure S1.** Analysis of stability and repeatability for the transcriptome sequencing with low amount of RNA. A. Pearson correlation between each library of different initial amounts of RNA. PR: pre-receptive; RE: receptive; PO: post-receptive B. Hierarchical clustering of the top 200 differential expressed genes among three sequential uterine fluid samples.


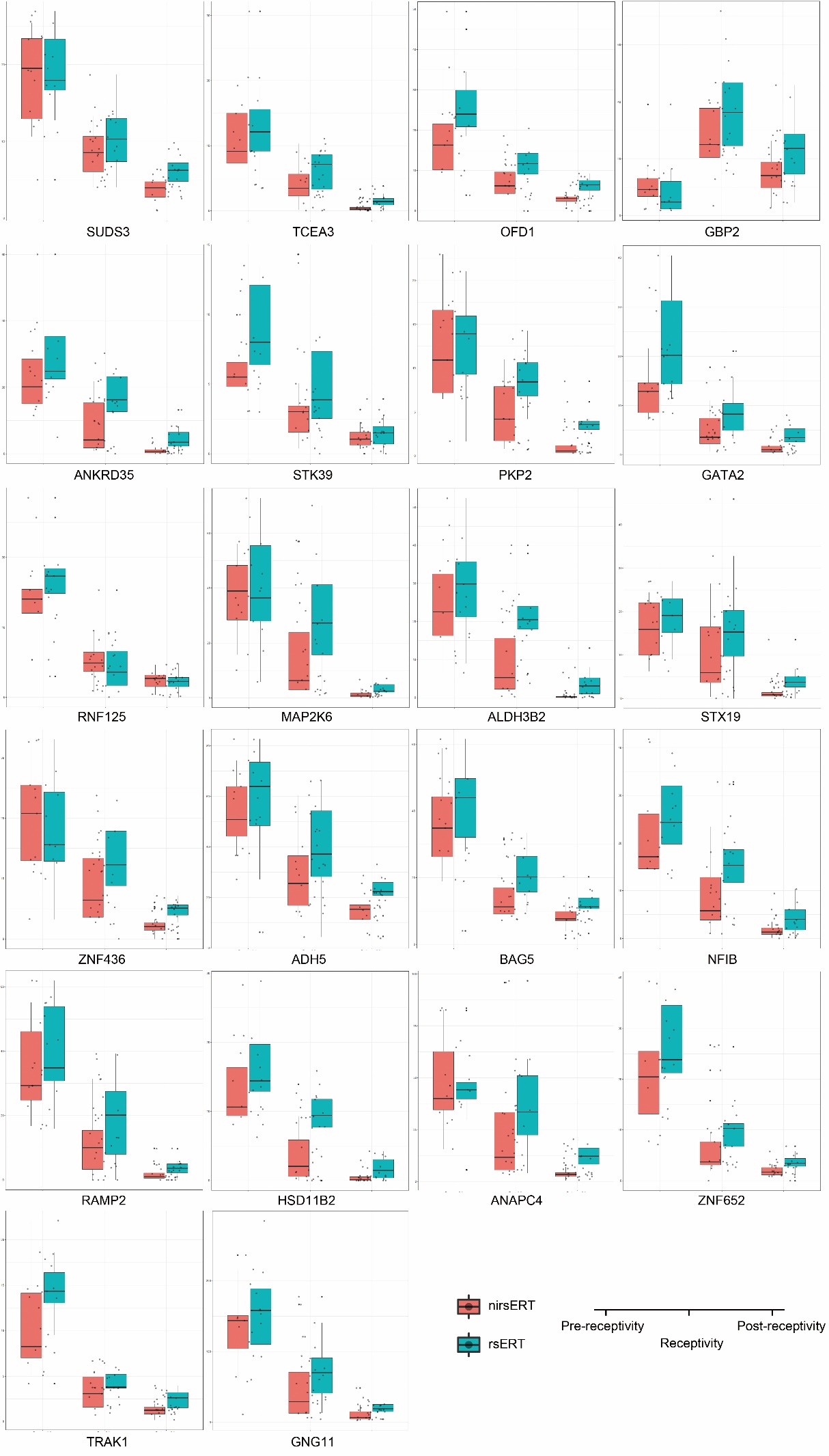


**Figure S2.** Expression pattern of 22 common markers between nirsERT and rsERT.


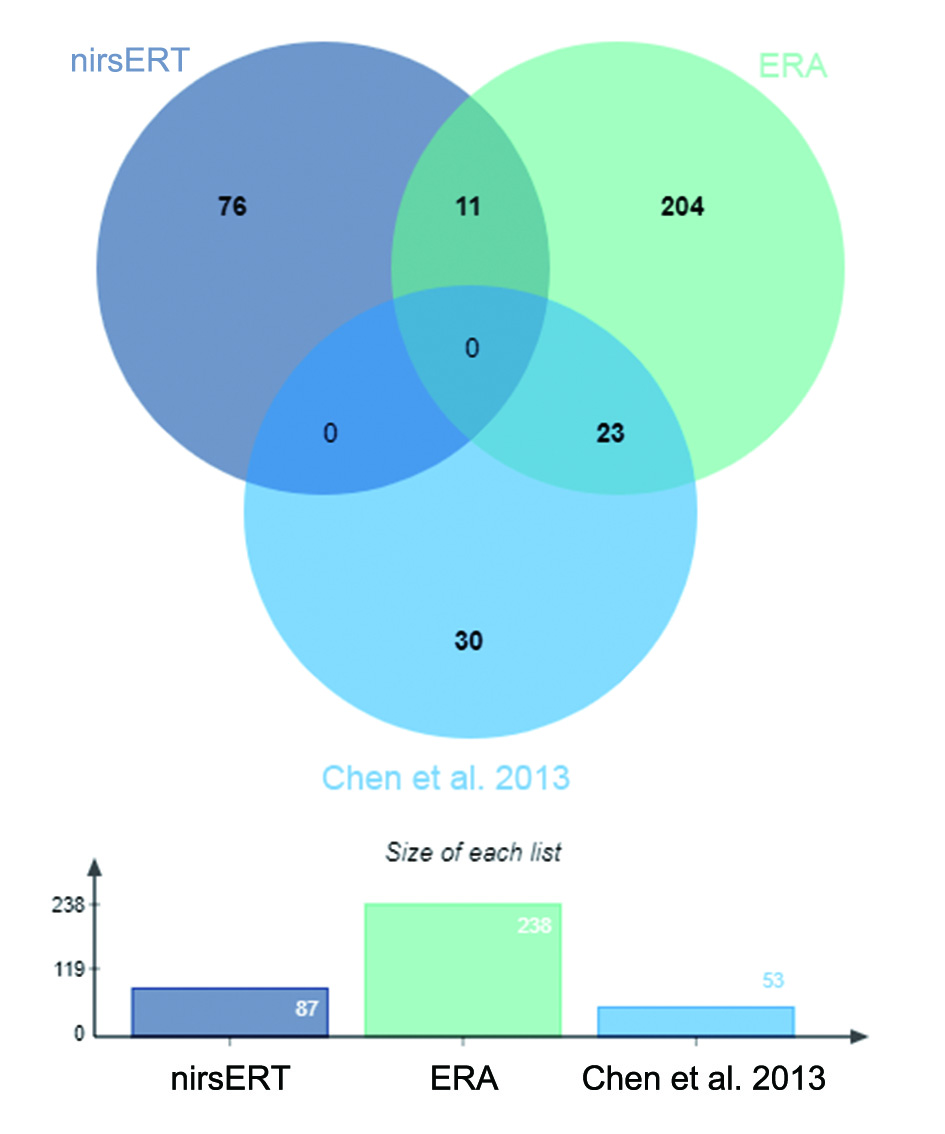


**Figure S3.** Venn diagram of predictive markers selected from three independent studies of endometrial receptivity. The Venn diagram is generated with jvenn (*2*).

**References**

1. C. Trapnell *et al.*, Transcript assembly and quantification by RNA-Seq reveals unannotated transcripts and isoform switching during cell differentiation. *Nat Biotechnol* **28**, 511-515 (2010).

2. P. Bardou, J. Mariette, F. Escudié, C. Djemiel, C. Klopp, jvenn: an interactive Venn diagram viewer. *BMC Bioinformatics* **15**, 293 (2014).
